# Ascorbate and Antibiotics, at Concentrations Attainable in Urine, Can Inhibit the Growth of Resistant Strains of *Escherichia coli* Cultured in Synthetic Human Urine

**DOI:** 10.3390/antibiotics12060985

**Published:** 2023-05-31

**Authors:** Carlos F. Amábile-Cuevas

**Affiliations:** Fundación Lusara, Mexico City 08810, Mexico; carlos.amabile@lusara.org; Tel.: +52-55-52195855

**Keywords:** ascorbic acid, urinary tract infection, nitrofurantoin, sulfamethoxazole, ciprofloxacin, gentamicin, *Escherichia coli*

## Abstract

There are conflicting reports on the antibacterial activity of ascorbate; all at concentrations much higher than the typical in human plasma, but that can be reached in urine. The effect of 10 mM ascorbate (in itself not inhibitory) along with antibiotics, was tested both in Mueller-Hinton broth (MHb) and in synthetic human urine (SHU), against resistant isolates of *Escherichia coli* from lower urinary infections. The activity of nitrofurantoin and sulfamethoxazole was higher in SHU than in MHb; minimal inhibitory concentrations (MICs) in SHU with ascorbate were below typical urinary concentrations. For other antibiotics, MICs were the same in MHb vs. SHU, with no effect of ascorbate in MHb; but in SHU with ascorbate, MICs of ciprofloxacin and gentamicin also went below reported urinary concentrations, with a lesser effect with norfloxacin and trimethoprim, and none with ampicillin. The effect of ascorbate was independent of oxygen and not related to the susceptibility of each strain to oxidative stress. Ascorbate oxidizes during incubation in SHU, and bacterial growth partially prevented oxidation. These results suggest that 10 mM ascorbate can enhance the inhibitory activity of antibiotics upon resistant strains in urine. Clinical experimentation with ascorbate–antibiotic combinations against urinary infections caused by resistant bacteria is warranted.

## 1. Introduction

Ascorbic acid (vitamin C) has a long and controversial history of reported antimicrobial capabilities. Perhaps the best-known example of this is the therapeutic effect of vitamin C upon viral respiratory diseases proposed by Linus Pauling in the 1970s, which was partially based on its alleged anti-viral activity in vitro [1] and that was explored even against COVID-19 [2]. Reports of the effects of ascorbate (ASC) on bacteria are diverse and conflicting, and have appeared for many years. For instance, ASC (1.7–5.4 mM) was found to have a bactericidal effect upon several uropathogens in urine, independent of pH [3]; the minimal inhibitory concentration (MIC) of ASC upon clinical isolates of *Pseudomonas aeruginosa* was in the 18.7–1233 mM range [4], but MIC_50_ and MIC_90_ of ASC upon clinical isolates of *P. aeruginosa* were reported to be 1.8 mM and 3.6 mM, respectively (although this seems to have been assessed using non-neutralized ascorbic acid solutions [5]), and ASC MICs for *Escherichia coli* and *P. aeruginosa* “standard strains” were 34 and 45 mM, respectively [6]; *Mycobacterium tuberculosis* seems particularly susceptible to ASC, with a MIC of 1 mM (contrasted with MICs of 16 and 32 mM for *P. aeruginosa* and *E. coli*, respectively, and >32 mM for *Staphylococcus aureus* and *Enterococcus faecalis* [7]); 57 mM ASC inhibits the planktonic growth of *Proteus mirabilis* [8]; the MIC of ASC upon clinical isolates of *Acinetobacter baumannii* was in the 3–6 mM range (although, surprisingly, the MIC for reference strain ATCC 1506 was 0.045 mM [9]).

ASC interaction with antibiotics and antibiotic resistance has also been documented: ASC had synergic effect with sulfonamides, trimethoprim, chloramphenicol, ampicillin, erythromycin and colistin in checkerboard assays on *P. aeruginosa* [4]; a 1-mM ASC treatment eliminates penicillinase plasmids from *S. aureus*, and reduces the MIC of penicillin and tetracycline [10,11]; a triple combination of ASC, apo-transferrin and imipenem was effective against carbapenem-resistant *A. baumannii* [9]; biofilm formation by *P. aeruginosa* was inhibited by ASC at 11–34 mM concentrations [6] and at 0.2–2 mM (possibly non-neutralized ASC; and also diminished piperacillin, ceftazidime, ciprofloxacin and gentamicin MICs [5]); but 2 mM ascorbate reduced the biofilm inhibitory activity of aminoglycosides gentamicin and amikacin [8]; addition of 10 mM ASC decreased the MIC of some antibiotics (e.g., ampicillin, tetracycline) and increased the MIC of others (e.g., erythromycin, ciprofloxacin) when testing *S. aureus*, several *Streptococcus* spp., and *E. coli* [12]. In non-PubMed-indexed journals reports are even more disparate (e.g., [13,14,15]).

The typical plasma concentration of ASC in humans is 0.05–0.07 mM [16]. This is well below most of the conflicting MICs mentioned above. However, ASC is readily excreted in urine, with urinary concentrations in the 0.1–16 mM range (3.5 mM average) when taking daily, 1-g oral ASC supplements [17]; higher doses, and/or intravenous administration may lead to even higher urinary concentrations, although there is no published data on this regard. This opens the possibility for the clinical use of some of the purported antibacterial capabilities of ASC against lower urinary tract infections (LUTI), alone or in combination with antibiotics, especially against those caused by antibiotic-resistant strains. Clinicians have used ASC against UTIs for many years aiming at “acidifying the urine” (which, in turn, is supposed to inhibit bacterial growth, but might do the exact opposite [18]); however, even at a 4-g daily dosage, ASC does not acidify the urine [19]. On the other hand, taking ASC supplements diminishes the risk of UTI by up to 40% [20]. To assess the possible interactions of ASC and antibiotics in vitro, resistant *E. coli* isolates from LUTI were grown in standard media (Mueller-Hinton broth, MHb) and in synthetic human urine (SHU [21]), in the presence of different combinations of ASC and antibiotics. This experimental design has the double purpose of combining both ASC and antibiotics at concentrations clinically attainable in an environment, SHU, more resembling an actual LUTI. A total of 10 mM ASC reduced the MIC of several antibiotics to levels that could be clinically relevant.

## 2. Results

ASC did not have inhibitory effects upon any of the strains tested (including ATCC 25922) up to 20 mM, either in MHb or SHU; further concentrations were not tested, as they would be clinically irrelevant.

### 2.1. Nitrofurantoin and Sulfamethoxazole Activity Was Affected Both, by SHU and ASC

MICs of nitrofurantoin and sulfamethoxazole decrease when assessed in SHU, and further diminish in the presence of ASC. MIC of nitrofurantoin upon five resistant strains was consistently reduced from 128 µg/mL to 32 µg/mL, comparing the values obtained in MHb and SHU, respectively. The presence of 10 mM ASC further reduced the MIC one to two dilutions, to 8–16 µg/mL (Table 1). The effect of ASC did not seem to be related to a phenotypic resistance to pro-oxidants, as the same, two-dilution reduction was attained in either “resistant” strains (2 and 16) or the “susceptible” one (12). The MIC of sulfamethoxazole was 1–2 dilutions lower in SHU than in MHb, in six out of seven strains tested, and further diminished 1–2 dilutions in the presence of 10 mM ASC (Table 1).

### 2.2. ASC Reduced the MIC of Fluoroquinolones in 1–2 Dilutions in SHU

MIC values of fluoroquinolones ciprofloxacin and norfloxacin were the same whether assessed in MHb or in SHU and were not changed by the presence of 10 mM ASC when in MHb. However, when growing in SHU, 10 mM ASC was capable of reducing the MIC of both fluoroquinolones in 1–2 dilutions (Table 2). Again, no correlation was observed between the effect of ASC and the level of “resistance” towards hydrogen peroxide and/or superoxide.

### 2.3. ASC Reduced the MIC of Gentamicin and Trimethoprim in 1–4 Dilutions in SHU

MICs of gentamicin and trimethoprim were the same either in MHb and SHU and were not affected by ASC when in MHb. However, when growing in SHU, gentamicin MICs were reduced by 1–2 dilutions, and trimethoprim MICs by at least 1–4 dilutions (Table 3).

### 2.4. Ampicillin MICs Did Not Change in any Experimental Condition

Strains 3, 5, 6, 7, 8 and 14 were tested with ampicillin; MIC for all of them were above 4096 µg/mL either in MHb or SHU, in the presence or absence of 10 mM ASC.

### 2.5. Dose-Response in Checkerboard Experiments

**Figure 1:**
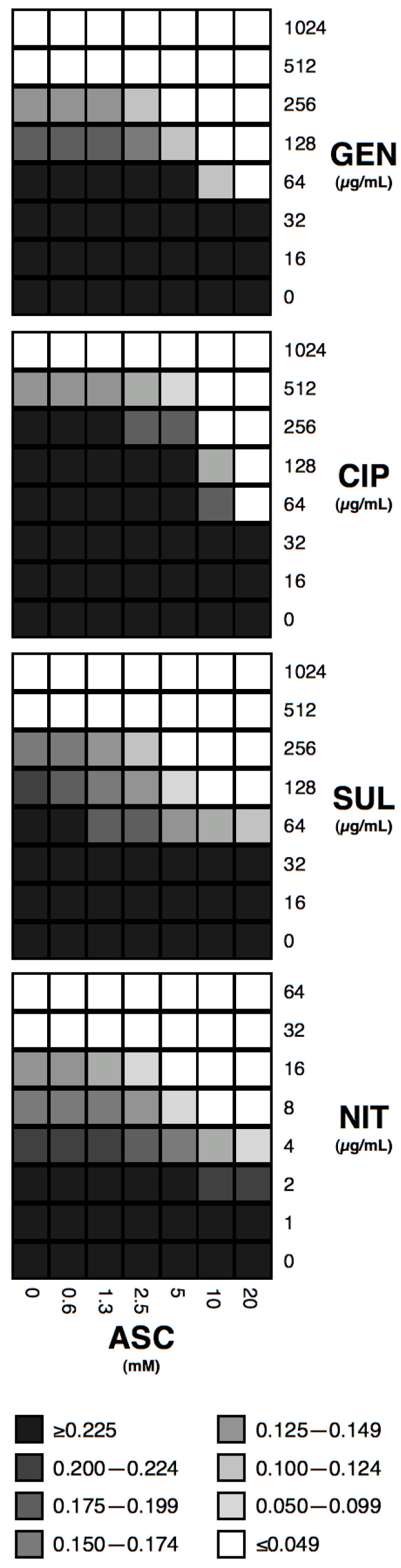
Checkerboard experiments with ASC and antibiotics. Combinations of ASC and gentamicin (GEN, upon strain 14), ciprofloxacin (CIP, upon strain 10), sulfamethoxazole (SUL, upon strain 10), and nitrofurantoin (NIT, upon strain 2) are shown. Grey scale is shown for OA_600nm_ of at least three independent experiments.

Checkerboard experiments upon strains most susceptible to ASC effect, show consistent dose-response effects for all four antibiotic-ASC combinations (Figure 1). For gentamicin and ciprofloxacin, a further reduction in MIC can be achieved at 20 mM ASC (to 64 µg/mL in both cases), but not for sulfamethoxazole nor nitrofurantoin.

### 2.6. The Effect of ASC on Antibiotic’s MIC in SHU Is Not Dependent on Oxygen

The same results as reported above were obtained when incubating plates or tubes that were sealed with an N_2_-CO_2_ atmosphere before incubation (except for gentamicin, which had no effect under anaerobic conditions). Growth was slower but still detectable after 24-h incubation; extending incubation to 36 h yielded similar growth than under aerobic conditions, and equal MICs.

### 2.7. ASC Is Oxidized in SHU, and Bacterial Growth Partially Prevents This Oxidation

After a 24-h incubation in SHU (35 °C, 250 rpm), the amount of ASC detectable by the 2,2′-dipyridyl method diminished from 10 mM to 3.1 mM (according to a calibration curve made with 2, 5, 7 and 10 mM fresh ASC added to fresh SHU); ASC in “ultra-pure” water incubated under the same conditions resulted in the loss of only 1.3 mM compared to ASC added immediately before testing. ASC 10 mM added to SHU without iron (SHU contains 5 µM FeSO_4_·7H_2_O) diminished to 6.1 mM after incubation. Bacterial growth partially prevented the loss of ASC to ~5.5 mM (depending on the strain). This was initially detected by a brownish color of wells where antibiotics prevented bacterial growth. The method using 2,4-dinitrophenylhydrazine yielded erratic results indicating interference of SHU (with or without iron) with the reactions.

## 3. Discussion

ASC is a particularly difficult chemical compound to work with: it oxidizes rapidly in aqueous solutions, especially in non-acidic conditions; it also can behave both, as an antioxidant, and as a pro-oxidant, depending on the concentration and the presence of other molecules, such as transition metal ions [22]. This is perhaps the reason behind the conflicting reports of antibacterial activity summarized in the Introduction. ASC is transported into *E. coli* cells by the *ulaABC* system and another yet unidentified transporter; but is not metabolized under aerobic conditions [23]. The interactions of ASC with antibiotics are further complicated by the possible generation of reactive oxygen species (ROS) by some antibiotics, especially those of bactericidal effect [24]. It is therefore conceivable that ASC may potentiate the activity of some antibiotics, acting as a pro-oxidant, while protecting against others, and acting as an antioxidant. While this has not been reported before for ASC-antibiotics combinations, it was for ASC and PQ or menadione, both redox-cycling compounds that generate intra-cellular superoxide radicals: while ASC protected *E. coli* against PQ toxicity (as well as vitamin E, another known antioxidant), it increased menadione-mediated cell death (while vitamin E protected against [25]). Also, agents that “mitigate” the effects of ROS (dipyridyl, DMSO) did reduce the toxicity of fluoroquinolones upon *E. coli* [26]. Antioxidant polyamines also protect *E. coli* against cefotaxime, netilmicin and levofloxacin [27]. Considering all these variables, disparate and paradoxical effects were expected in this project; it was also the reason for selecting the SHO model as being of a more controlled composition compared to typical culture media, and also more closely related to clinical conditions.

SHU has been proposed as a standardized way to simulate the actual conditions of microbial growth during UTI and bacteriuria, in vitro [21,28]. It yields more reliable results than pooled urine [29]. The effect of antibiotics upon bacterial growth has been tested before in different formulations of SHU (e.g., [30,31,32]) providing a satisfactory medium for in vitro modeling of antimicrobial treatment of IVUs. While SHU lacks some of the organic components of real human urine, such as hormones, peptides, and cell debris, it is clearly closer to it than the typical culture media used for antibiotic susceptibility tests (e.g., Mueller Hinton broth is a mixture of powdered beef extract and casein acid hydrolysate). Along with pharmacokinetic factors that make antibiotics more or less concentrated in urine than in other body fluids [33], the great divergence between urine and culture media could be behind the “little to no clinical predictive value” of typical in vitro susceptibility tests in UTIs [34]. Here, comparing the inhibitory activity of antibiotics alone, either in MHb or SHU, resulted in interesting differences: while MICs of ampicillin, ciprofloxacin, gentamicin, and trimethoprim did not change in either medium, MICs of sulfamethoxazole and nitrofurantoin were lower in SHU than in MHb, especially for the latter. The clinical consequences of this disparity should be explored: perhaps these antibiotics are more effective in urine than in other fluids, which could explain why antibiotic therapy of UTIs is successful despite the causative bacteria being deemed resistant, in addition to classical pharmacokinetic considerations [33].

Ciprofloxacin and norfloxacin were selected, not only because they are typical fluoroquinolones used in UTIs, but also for the diverging relevance ROS have in their antimicrobial activity: while norfloxacin strongly depends on the generation of ROS to achieve bacterial killing, ciprofloxacin seems more dependent on the “canonical” mechanism of action, i.e., DNA fragmentation after inhibition of gyrases [35]. This difference is not reflected in different MICs of these fluoroquinolones in O_2_^−^ and/or H_2_O_2_ “resistant” strains, compared to “susceptible” or average ones (Table 2). ASC seems to reduce further the MIC of norfloxacin than that of ciprofloxacin, possibly by potentiating the intracellular generation of ROS by the antibiotic; antithetically, the O_2_^−^/H_2_O_2_ “resistant” strain (3) was more affected by ASC than the O_2_^−^/H_2_O_2_ “susceptible” strain (12). Furthermore, the effect of ASC was independent of the presence of oxygen, indicating that it is not dependent on the generation of ROS. MIC reduction by ASC was observed both, with bacteriostatic (nitrofurantoin, sulfamethoxazole) and bactericidal (fluoroquinolones, gentamicin) antibiotics, which could suggest that the effect is not related to the proposed generation of ROS by bactericidal agents [36]. However, both nitrofurantoin and sulfamethoxazole do elicit anti-oxidant responses in *E. coli*, activating the *soxRS* regulon the former [37], and the latter a family of pteridines, called “colipterins”, having anti-oxidant capabilities [38], suggesting that oxidative stress is also related to the action of these drugs.

During all the MIC and checkerboard experiments, a brownish color in the SHU supplemented with ASC was observed when bacterial growth was absent, either as non-inoculated solutions, or in those where growth was inhibited by antibiotics. This was observed before at ASC concentrations above 2 mM (which were inhibitory under the conditions reported there [23]). There are several methods for measuring ASC concentrations [39]; we attempted two of them, one using 2,2′-dipyridyl and another using 2,4-dinitrophenylhydrazine. As the latter yielded conflicting results, indicating interference by components of SHU, the former was used here. As the 2,2′-dipyridyl method measures the formation of a ferrous-dipyridyl complex after the reduction of ferric ion to ferrous ion by ASC, it detects non-oxidized ASC (unless oxidized ASC is reduced with dithiothreitol [40]). ASC dissolved in “ultra-pure” water was fairly stable, with a 13% loss after 24-h incubation; when dissolved in SHU without iron, 39% was lost, and in SHU with iron, the loss was 69%. Bacterial growth in iron-containing SHU diminished the loss of ASC to 45% (bacterial growth is minimal in SHU without iron). It is possible that bacterial growth rapidly quenched available iron in SHU, thus preventing further ASC oxidation; Interestingly, a “total antioxidant status” (namely, the ability of a fluid to prevent a colored reaction initiated by hydroxyl radicals) was reported to be higher in the urine of UTI patients than in infection-free controls [41]. This could indicate that overall conditions observed in vitro in the experiments reported here are also found in vivo during UTIs.

Urinary concentrations of antibiotics are difficult to assess, because of the irregular timing of sample availability, among other things. Some of the published data are summarized in Table 4. Comparing the MICs observed in SHU w/wo ASC, some predictions can be drawn:

(a) ampicillin would not be effective, as MICs are not affected by the presence of ASC and are always well above maximum urinary concentrations; trimethoprim MICs, while being reduced by the presence of 10 mM ASC, are still above urinary concentrations;

(b) nitrofurantoin alone should be effective even against resistant isolates, as MICs in SHU are below urinary concentrations; and ASC should enhance this activity even further;

(c) six out of eight ciprofloxacin-resistant isolates should be inhibited at urinary concentrations reached after taking a 1-g extended-release tablet, and in the presence of 10 mM ASC; but only 3/8 should be inhibited by norfloxacin after taking a 400-mg tablet in the presence of ASC;

(d) six out of seven sulfamethoxazole-resistant isolates could be inhibited by sulfamethoxazole-ASC, but only considering the highest value reported in the urine (600 µg/mL), corresponding to 500 mg BID; and

(e) gentamicin-ASC could be effective against three out of four resistant isolates in the presence of ASC and after a 160-mg intramuscular dose (or even all isolates, taking the value reported for a 1.6 mg/kg intramuscular dose).

Checkerboard experiments were performed to look for paradoxical effects, which ASC might have at different concentrations. However, effects described a dose-response behavior, and 10 mM ASC achieved the best effect within the concentration values reported previously in urine. Overall, ASC at concentrations achievable in urine after taking at least a 1-g vitamin C oral supplement, seems likely to restore the inhibitory effects of most antibiotics tested here upon resistant strains. Of course, these are only theoretical predictions based on the results of in vitro experiments shown here and must be clinically tested, but these results should indeed encourage such clinical trials.

While large quantities of bacteria exist in the urine during a LUTI, there is also an intracellular component of the infectious process [54]. When treating urothelial cells in cultures that have been intracellularly infected with *E. coli*, with antibiotics at the typical urinary concentrations found in a medicated patient, there are significant differences in antimicrobial activity. For instance, fluoroquinolones and nitrofurantoin eradicate more than 90% of the intracellular bacteria, while beta-lactams and aminoglycosides have no effect [43]. Intracellular ASC concentrations vary widely, from 0.05 mM in erythrocytes to 10 mM in neurons; sodium-ascorbate co-transporters (SVCT1), such as those in the apical side of epithelial cells, can cause large influxes of ASC when in the presence of high ASC concentrations. ASC can also cross biological membranes by passive diffusion [22]. Once inside the cell, ASC is trapped because of its electrical charge at physiological pH [55]. It is therefore conceivable that reaching a 10 mM ASC concentration in urine could result in similar intracellular concentrations in the urothelium, therefore allowing for a similar effect to the one reported here in vitro. Along with effective intracellular antibiotics, such as nitrofurantoin, ciprofloxacin, and, to a lesser extent, sulfamethoxazole, ASC could act as an adjuvant against infections caused by resistant bacteria. Clinical testing is warranted.

## 4. Materials and Methods

### 4.1. Bacterial Strains

*E. coli* strains used (Table 5) were isolated from urine samples of female patients suffering from LUTI, over several years for surveillance purposes. All isolates were identified by standard biochemical methods, antibiotic susceptibility was initially assessed by disc diffusion on Mueller-Hinton agar plates, following CLSI guidelines [56], and kept in liquid media containing 25% glycerol under liquid nitrogen. Strains were selected based on relevant resistance phenotypes. *E. coli* ATCC 25,922 was used for routine control of antibiotic susceptibility methods.

### 4.2. Assessment of Pro-Oxidant Susceptibility

The overall susceptibility to pro-oxidant compounds, namely hydrogen peroxide (H_2_O_2_) and paraquat (PQ, a redox-cycling agent known to generate intracellular superoxide radicals) was measured by disk diffusion, using filter paper discs containing either 400 µg of PQ (Sigma), or 8.8 µmol of H_2_O_2_ (Sigma), placed on top of LB agar plates previously inoculated with each strain. The diameter of inhibitory zones was measured after a 35 °C/overnight incubation [57]. From a previous survey, average ± one standard deviation inhibitory zones for PQ discs were 12–18 mm, with those strains producing inhibitory zones of 11 mm or less being considered “resistant”, and those of 19 mm or more is considered “susceptible”; and for H_2_O_2_, average ± one standard deviation was 23–28 mm, those of 22 mm or less were considered “resistant”, and those of 29 mm or more were considered “susceptible” [58].

### 4.3. SHU, ASC and Antibiotics Solutions

For all experiments, a 1 M sodium ascorbate (Sigma) stock solution was used, dissolving the salt in “ultra-pure” water (distilled water passed through a Barnstead EASYpure RF system up to 18.2 MΩ/cm), filter-sterilized, aliquoted and stored under liquid nitrogen for no longer than a week and thawed only once before use. SHU supplemented with casamino acids was prepared as reported by Ipe et al. [21]. Stock solutions of antibiotics (ampicillin, sulfamethoxazole, trimethoprim, from Sigma; norfloxacin form USP, gentamicin, a kind gift from Schering-Plough; ciprofloxacin, a kind gift from Bayer) were prepared as recommended by CLSI, except for nitrofurantoin (Sigma), which was dissolved in DMSO to 50 mg/mL.

### 4.4. MIC Assays with/without ASC

MICs of ampicillin (AMP), ciprofloxacin (CIP), gentamicin (GEN), nitrofurantoin (NIT), sulfamethoxazole (SUL), and trimethoprim (TMP) were assessed first in MHb by microdilution following CLSI guidelines, in 96-well plates, incubated statically, to confirm resistance phenotypes obtained by disc diffusion; and then in MHb and SHU, with or without 10 mM ASC, incubated at 35 °C for 24 h shaken at 250 rpm, both in tubes (1 mL) or 96-well plates (100 µL). For anaerobic incubation, plates or tubes were prepared and sealed within a glove bag previously filled with a 9:1 mixture of nitrogen and carbon dioxide, before incubating under the conditions stated above.

### 4.5. Checkerboard ASC/Antibiotic Assays

Checkerboard experiments [59] with ASC (0.62–20 mM) and antibiotics were performed in SHU upon the strains that showed the maximal effect in the ASC 10 mM assays: strain 2 for nitrofurantoin (1–64 µg/mL), strain 10 for sulfamethoxazole (16–1024 µg/mL) and for ciprofloxacin (16–1024 µg/mL), and strain 14 for gentamicin (16–1024 µg/mL).

### 4.6. ASC Measurement

ASC concentration after incubation in SHU was assessed by the methods using 2,4-dinitrophenylhydrazine, and 2,2′-dipyridyl, as described before [39].

## Figures and Tables

**Table 1:** Susceptibility to oxidative stress, and MICs (µg/mL) of nitrofurantoin and sulfamethoxazole upon resistant *E. coli* strains, in Mueller-Hinton broth and synthetic human urine, with and without 10 mM ascorbate.

			MHb		SHU		MHb		SHU	
Strain	O_2_^−^	H_2_O_2_	NIT	NIT/ASC	NIT	NIT/ASC	SUL	SUL/ASC	SUL	SUL/ASC
1	R	R	128	64	32	16				
2	R	R	128	32	32	8				
8	A	A	128	32	32	16	2048	1024	1024	512
9	A	A					2048	1024	512	256
10	A	A					1024	1024	512	128
11	R	A					2048	1024	1024	256
12	S	S	128	32	32	8	1024	512	1024	256
13	A	A					1024	1024	512	128
14	A	A					512	256	512	128
16	R	R	128	32	32	8				

O_2_^−^, superoxide (paraquat disc): strains were considered “average” (A) with a diameter of inhibition zone (DIZ) of 12–18 mm, “resistant” (R) with DIZ ≤ 11 mm, and “susceptible” (S) with DIZ ≥ 19 mm; H_2_O_2_, hydrogen peroxide; (A) with DIZ of 23–28 mm, (R) with DIZ ≤ 22 mm, (S) with DIZ ≥ 29 mm (see Material and Methods). MHb, Mueller-Hinton broth; SHU, synthetic human urine. NIT, nitrofurantoin; SUL, sulfamethoxazole; ASC, ascorbate 10 mM. Results of at least three independent experiments.

**Table 2:** Susceptibility to oxidative stress, and MICs (µg/mL) of fluoroquinolones upon resistant *E. coli* strains, in synthetic human urine, with and without 10 mM ascorbate.

Strain	O_2_^−^	H_2_O_2_	CIP ^a^	CIP/ASC	NOR ^a^	NOR/ASC
3	R	R	1024	512	>1024	256
4	A	R	>1024	512	>1024	512
5	R	A	>1024	>1024	>1024	>1024
6	R	A	1024	512	>1024	1024
10	A	A	1024	256	>1024	256
12	S	S	>1024	1024	>1024	>1024
14	A	A	>1024	512	>1024	1024
15	A	A	>1024	512	>1024	256

^a^ ciprofloxacin and norfloxacin at 2048 µg/mL precipitated during incubation. O_2_^−^, superoxide (paraquat disc); H_2_O_2_, hydrogen peroxide: strains were considered “average” (A), “resistant” (R) or “susceptible” (S) as in Table 1. CIP, ciprofloxacin; NOR, nofloxacin; ASC, 10 mM ascorbate. Results of at least three independent experiments.

**Table 3:** Susceptibility to oxidative stress, and MICs (µg/mL) of gentamicin and trimethoprim upon resistant *E. coli* strains, in synthetic human urine, with and without 10 mM ascorbate.

Strain	O_2_^−^	H_2_O_2_	GEN	GEN/ASC	TMP	TMP/ASC
3	R	R	512	128		
8	A	A	256	128	>2048	512
9	A	A			>2048	1024
10	A	A			>2048	128
11	R	A			>2048	256
12	S	S	512	256	>2048	1024
13	A	A			>2048	1024
14	A	A	512	128	>2048	1024

O_2_^−^, superoxide (paraquat disc); H_2_O_2_, hydrogen peroxide: strains were considered “average” (A), “resistant” (R) or “susceptible” (S) as in Table 1; (A) with halos of 23–28 mm, (R) with halos ≤ 22 mm, (S) with halos ≥ 29 mm. GEN, gentamicin; TMP, trimethoprim; ASC, 10 mM ascorbate. Results of at least three independent experiments.

**Table 4:** Antibiotic concentrations (µg/mL) in urine after standard dosing.

Ref.	AMP	CIP	GEN	NIT	NOR	SUL	TMP
[42]	160–700	>2	400–500	25–300	168–417	100–100	70–100
Kucers ^a^	250–1000	20–387 ^b^	40–50	50–250	478	40–320	19–130
[43]	-	2–3	400–800	300–500	300–500	100–300	66–200
other		268 ^c^ 892 ^d^	164–225 ^e^	70 ^f^			

^a^ chapters from Kucers’ The use of antibiotics: AMP, [44]; CIP, [45]; GEN, [46]; NIT, [47]; NOR, [48]; SUL and TMP, [49]. ^b^ values for the unlikely single dose of 200 mg IV. ^c^ for 500 mg, orally [50]. ^d^ for 1000 mg, extended release [51]. ^e^ [52]. ^f^ [53].

**Table 5:** Phenotypic resistance of selected *E. coli* isolates.

Strain No.	AMP	AMC	CTX	SUL	CIP	GEN	NIT	Used for Assays with:
1	R	R		R			R	NIT
2	R	R	R	R	R		R	NIT
3	R				R	R		Flu, AMP, GEN
4	R	R	R	R	R			Flu
5	R			R	R			Flu, AMP
6	R			R	R			Flu, AMP
7	R			R				AMP
8	R	R	R	R	R	R	R	NIT, SUL, AMP, GEN
9				R	R			SUL
10	R	R		R	R			SUL, Flu
11	R	R		R				SUL
12	R	R		R	R	R	R	NIT, SUL, Flu, GEN
13	R	R		R				SUL
14	R			R	R	R		SUL, Flu, AMP, GEN
15	R	R		R				Flu
16				R	R		R	NIT

Results from disc diffusion tests; empty cells indicate susceptibility. AMP, ampicillin; AMC, amoxicillin-clavulanate; CTX, cefotaxime; SUL, sulfonamide; CIP, ciprofloxacin; GEN, gentamicin; NIT, nitrofurantoin, Flu, fluoroquinolones.

## Data Availability

Not applicable.
